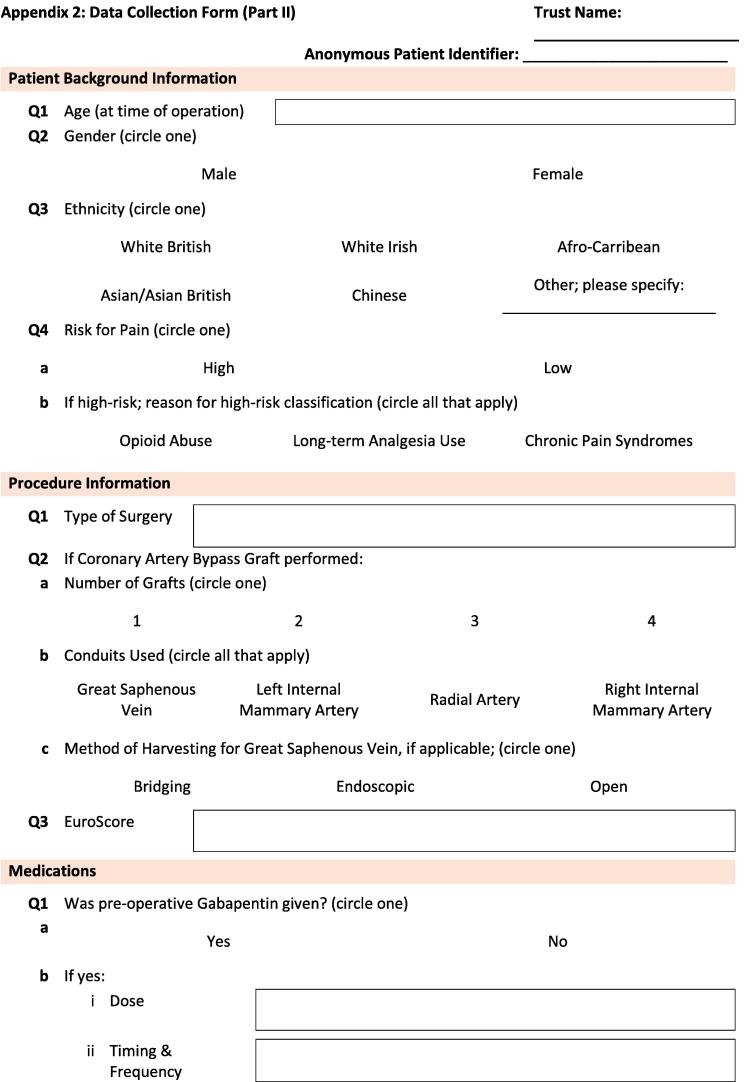# Optimising pain management protocols following cardiac surgery: A protocol for a national quality improvement study

**DOI:** 10.1016/j.isjp.2018.12.002

**Published:** 2019-01-11

**Authors:** S. Jayakumar, M. Borrelli, Z. Milan, G. Kunst, D. Whitaker

**Affiliations:** a Department of Cardiothoracic Surgery, King’s College Hospital, United Kingdom; b Division of Plastic Surgery, Stanford University School of Medicine, United States; c Department of Anaesthesia, King’s College Hospital, United Kingdom

## Abstract

Pain following cardiac surgery is a multifaceted phenomenon resulting from a number of mechanisms. High-levels of post-operative pain are associated with cardiovascular and respiratory complications and adequate pain management is crucial for enabling fast recovery. However, adequate pain control is complex, a challenge that stems from a combination of poor reporting of pain, significant variation amongst patients and the side-effects of strong, particularly opioid, analgesics. An initial audit at our hospital demonstrated high-levels of post-operative pain following cardiac surgery and a protocol was therefore devised by the anaesthetic department for cardiac surgical pain management. The protocol stratified patients into high- or low-risk of pain based on the presence of risk factors for pain and utilised a combination of pre-operative one-off dose of gabapentin, intra-operative opioid infusion and post-operative multimodal analgesia with paracetamol, weak and strong opioids. Additionally, patients at high-risk of pain also received patient controlled analgesia. Use of this protocol was associated with improved pain scores on the first three post-operative days. We have devised this study to test for reproducibility of the benefit experienced at our hospital at a larger multicentre level. After acquiring pre-existing post-operative pain management strategies through an initial survey, local study leads will undertake a baseline audit. Local study leads will then lead a 4-week period of protocol implementation. Trusts with official pain management protocols will be given the option to re-circulate their pre-existing protocols. Subsequently, pain scores during post-operative days 1–3 will be re-audited.

## Highlights

• Severe pain is associated with tachyarrhythmias, shallow breathing and poor recovery.
• Our protocol was effective at reducing post-cardiac surgery pain in a single centre.
• It consists of pre-operative gabapentin and dividing patients based on risk of pain.
• High-risk group receive PCA along with paracetamol and codeine given to all patients.
• Centres will undertake a baseline audit, then implement a protocol and re-audit pain.

## 1 Introduction

Pain following cardiac surgery is common, and, despite being largely avoidable, is moderate to severe in up to 75% of patients [1]. Pain can prolong hospital stays and cause significant morbidity including psychology distress and in some cases, chronic pain.

### 1.1 Mechanisms of pain

Acute pain following cardiac surgery is multifactorial in aetiology. Skin incision, dissection, sternal retraction, preparation of the internal mammary artery graft, placement of chest drains, endotracheal intubation and sternal wires all directly injure the tissue and instigate the release of a panel of pro-inflammatory mediators, including nitric oxide and cytokines. These mediators activate afferent nociceptive fibres and cause nociceptive pain. Nociceptive pain can be further exaggerated by the inflammation inherent with cardiopulmonary bypass and anaesthetic drugs [2]. Additionally, sternal retraction, especially during harvesting of the internal mammary artery, can dislocate and fracture ribs, and is a leading cause of musculoskeletal pain [3]. Misalignment of the replaced sternum with associated ribs can result in costochondritis [4]. Pleuritic pain may result from the placement of intercostal chest drains. In the immediate post-operative period, incisional and traumatic injury is the main source of pain, but as it subsides, musculoskeletal pain predominates [5]. Occasionally, pain may persist for prolonged periods following surgery, with 35% of patients reporting presence of persistent thoracic pain one year post cardiac surgery [6]. Chronic pain most often arises from traumatic or inflammatory nerve injury which results in neuropathic pain [7]. Sternal wires can also trigger an exaggerated fibrotic response leading to excessive inflammation and entrapment of sensory nerves.

### 1.2 Adverse effects of pain

High levels of post-operative pain are associated with numerous consequences detrimental to recovery. Pain inhibits satisfactory coughing and deep breathing, and patients in pain breathe more rapidly and less deeply. This results in mucus accumulation and puts patients at an increased risk of atelectasis and pneumonia, consequently prolonged hospital stays, mechanical ventilation, and antibiotics [8]. Additionally, patients in pain are less likely to be mobile, sit upright and comply with their physiotherapy. In addition to promoting atelectasis, this may exacerbate disuse induced muscle atrophy and increase the time taken to return to a level of mobility satisfactory for discharge [9].

Pain also activates the sympathetic nervous system (SNS) and stimulates the hypothalamic–pituitaryadrenal axis (HPA). Increased release of adrenaline elevates blood pressure, increases heart rate, and induces a hyperglycaemic state [10]. This unfavourable cardiovascular state can promote arrhythmias, including atrial fibrillation (AF), and increase myocardial oxygen consumption, predisposing patients to ischaemic events [11]. The incidence of postoperative AF ranges from 29% to 63% depending on the cardiac surgical procedure, and may warrant a readmission to cardiac intensive care or the high dependency unity for close monitoring [12], [13], [14].

Pain is also a substantial source of anxiety and distress for patients, worsening sleep and leading to exhaustion and low mood. Severe or prolonged acute pain is a risk factor for the development of chronic pain, with 21–55% of patients developing chronic pain syndromes after cardiac surgery [8]. This may then have serious implications for the patient’s quality of life and is also a strong risk factor for depression, with up to 30% of patients with chronic pain developing depression [15].

### 1.3 Management of pain

Post-operative pain is frequently undertreated. Challenges in adequately treating pain stem from numerous factors, these include the under-reporting of pain, inter-individual variation in pain thresholds and complications of over-analgesia which can reduce consciousness, impair breathing and cause nephrotoxic effects. Furthermore, evidence has shown the importance of using multi-modal analgesia in treating post-operative pain [16]. This means that a combination of analgesics including neuropathic agents, opioids, paracetamol and/or non-steroidal anti-inflammatory drugs (NSAIDs) may enhance recovery and facilitate early mobilisation as well as decreased opioid usage and fewer side-effects [17]. Optimal multi-modal regimens are however difficult to achieve.

Opioids are usually the initial primary analgesia used following cardiac surgery and are particularly effective for pain experienced at rest. However, they have a narrow therapeutic window with numerous adverse effects in higher doses, including nausea, vomiting, pruritus, constipation and urinary retention. Large doses of opioids may also result in respiratory complications, decreased mobility due to increased drowsiness, and lead to addiction. Opioids can exacerbate renal failure and trigger post-operative ileus. Therefore, whilst they are very effective, they must be used with caution and for the minimum time that is necessary [18], [19]. Patient controlled analgesia (PCA), however, allows for closer matching of opioid administration to the level of pain by the patient, whilst minimising adverse effects, and therefore improves pain control. NSAIDs may also be used after surgery, though, are associated with an increased risk of post-operative bleeding, and precipitate acute kidney injury, particularly in patients undergoing cardiopulmonary bypass (CPB) [20], [21]. Paracetamol (acetaminophen) is also a widely used analgesic with a fairly safe drug profile in cardiac patients at therapeutic levels. It is often given intravenously in the first few days following surgery to maximise plasma drug concentrations. Finally, neuropathic agents, including gabapentin and pregabalin have been recently described as an effective adjuvant analgesic when started pre-operatively [22], [23].

### 1.4 Audit

The anaesthetic department at King’s College Hospital (KCH) found pain levels following cardiac surgery were unacceptably high with average scores of 4/10 on a Likert scale on an initial audit (audit cycle 1; AC1). A pain management protocol was devised (table 1). This protocol was created by the cardiothoracic anaesthetic team at King’s College Hospital based on clinical experience of post cardiac surgical pain in different patient populations. The protocol specified was devised for use by the clinical team in cardiac recovery, HDU and the cardiac surgery recovery ward until discharge. This protocol classified patients as high- or low-risk of pain. High-risk patients were defined as those who had a history of opioid abuse, previous chronic pain conditions, or long term analgesic use. Based on evidence from 4 recent RCTs [22], [24], [25], [26], the team suggested all patients be given a pre-operative dose of Gabapentin as an adjuvant to typical analgesics. The protocol also specified that pain levels should be measured as usual by the nursing team. Unacceptable levels of pain were classified as ≥4/10 at rest, and ≥8/10 on moving or coughing [8]. The protocol required health care professionals to call the pain team if pain scores exceeded 8.

**Table 1:** King’s College Hospital Cardiac Surgical Pain Management Protocol.

	High-risk Defined as: previous IVDU, chronic pain or long-term analgesic use	Low-risk Defined as: all non-high-risk patients
*Pre-operative*		
Gabapentin	600 mg STAT	

*Intra-operative*		
Opioid infusion (morphine or fentanyl as per patient profile)	Yes	Yes

*Post-operative*		
Opioids	Morphine infusion until extubation → convert to tramadol	
Paracetamol + codeine	Regularly or as necessary	
Patient controlled analgesia	Yes	No
Call acute pain team	If pain score ≥8	

Two subsequent audits, 6 months apart (AC2 and AC3), were conducted to evaluate adherence to the protocol and improvement in pain scores following cardiac surgery with a conventional midline sternotomy [27]. Pain scores were measured at rest, on moving and coughing during the first 3 postoperative days (POD1, POD2, POD3) using the numerical rating scale (0–10). The audits found the protocol was not adhered to in the majority of patients. Pre-operative gabapentin was only given in 50% patients. The pain team was only called twice in AC2 despite 26 incidences of pain scores >8 in AC2. Therefore, information about the protocol was circulated through the department in a systematic manner, through audit presentation, posters and email, to improve departmental awareness of pain and the protocol following AC2. AC3 found pain scores were significantly reduced (12 incidences of pain scores >8). However, no improvement in protocol adherence was found on measures of protocol adherence recorded. This improvement in pain scores was therefore hypothesised to result from increased awareness and attention to pain and the pain protocol by all staff, resulting in more frequent pain scoring and rapid analgesia delivery. It is possible audit protocol adherence was improved, but on an un-recorded measure of audit adherence. On observation patients received as necessary (PRN) medications more frequently and at lower thresholds of pain. The nursing team could have also been recording pain scores more frequently. This highlights the importance of the multidisciplinary team in pain management. Both audits compared pain between patients high- and low-risk of pain, and showed a non-significant increase in rest pain in high-risk patients. Interestingly, both audits also showed a single dose of pre-operative gabapentin made no difference to pain scores in both the cycles of the audit.

Given the possible substantial benefit to pain levels in this institution, and no current national guidance for pain following cardiac surgery, a multicentre audit was designed: 1) to assess whether this protocol, across a wider demographic of patients and increased number of patients, can be beneficial in all cardiac centres; 2) whether it is useful to distinguish between high- and low-risk patients, and, 3) whether a pre-operative dose of gabapentin has any effect on post operative pain. Additionally, this study aims to measure the previously unrecorded indexes of pain protocol adherence, specifically, frequency of pain recording per day, and the frequency at which PRN pain medication was given.

### 1.5 Study design & reasoning

This study will be conducted as a multicentre national study with the aim of including all the National Health Service (NHS) operated cardiac surgical centres in the United Kingdom. The reasoning behind this study is to test for reproducibility of the benefit of the initial single-centre pilot at a larger multicentre level.

### 1.6 Aims

The aims of this study are to improve pain management across UK Cardiac Surgical Centres and create a standardised cardiac surgical pain protocol stratified based on the risk factors for developing pain.

### 1.7 Objectives

The primary objectives are:

• To investigate the current strategies for pain management across different UK centres.
• To assess the effectiveness of current protocols.
• To understand current levels of unacceptable pain.

Data will be collected on current strategies including use of any protocols to enable a comparison between different protocols to elicit the most effective modes of pain management. This analysis may also provide further factors contributing to risk of developing pain and allow us to quantify the importance of various high-risk factors.

The secondary objective is to replicate the findings of this audit at this single tertiary centre with a larger data set, across a wider patient population, to elicit both, effectiveness of pain management and the extent of unacceptable levels of pain post-operatively across cardiac centres in the UK which can highlight shortcomings in current postoperative pain management. Implementing the protocol developed at KCH across trusts and including a large number of patients from varying backgrounds may also demonstrate subtleties in demographics and inter-individual variations that affect severity of pain.

### 1.8 Hypotheses

It was hypothesised that pain in post cardiac surgery across the UK is unacceptably high. It was hypothesised that trusts with an existing post-operative cardiac surgical pain management protocol have lower levels of post-operative pain amongst patients, particularly trusts that stratify management based on the patient’s risk of pain. It was also hypothesised that patients with risk factors for pain will have higher levels of pain.

## 2 Methods

### 2.1 Registration

This is a prospective multi-centre cohort study. This study will be registered as a multi-centre study through the integrated research application system (IRAS) to obtain ethical approval. All NHS cardiac centres in the UK will be identified through the Society of Cardiothoracic Surgeons (SCTS) database. Power analysis, based on previous audits, suggested a minimum of 50 patients per trust would be required to provide statistical significance.

### 2.2 Data collector recruitment

Data collection will be conducted at each registered NHS cardiac centre by up to three assigned trust medical students. The number of students per centre will depend on cardiac centre patient load. Medical students will be recruited through the Undergraduate Cardiovascular Research Network (UCRN). Each student will apply for research passport at their centre enabling them to collect data at their assigned trust and will have a local consultant lead supervising the study with whom they will liaise to obtain local trust approvals for the audit. The cardiac surgery consultant and cardiac anaesthetist at KCH will be the chief investigator (CI).

### 2.3 Inclusion/exclusion criteria

All patients undergoing cardiac surgery with a midline sternotomy at each trust over the age of 18 years will be considered as per the inclusion/exclusion criteria (Table 2).

**Table 2:** Inclusion/exclusion criteria.

Inclusion criteria	Exclusion criteria
• All patients ≥18 years undergoing a midline sternotomy	• Mini-sternotomy (defined as a sternotomy not extending the entire thoracic cage)
• Chest drains and vein harvest used in conjunction may be included	• Other concurrent chest incisions (e.g. endoscopic incisions, thoracotomy incisions)

### 2.4 Data collection

**Fig. 1:**
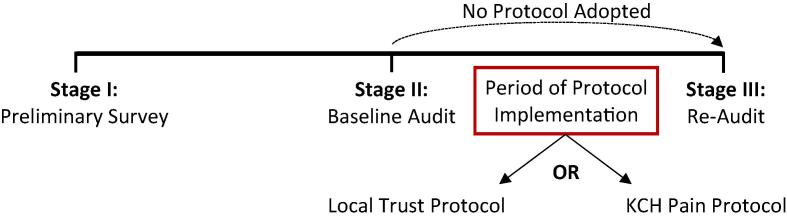
Study overview.

Data collection will take place in three stages (Fig. 1):

• The first stage will involve a preliminary survey to gather data on the cardiac surgical post-operative pain protocol in use at each participating trust. All protocols will be later analysed for their content.
• The second stage is the baseline audit, where data are collected over an 8-week period on pain scores prior to any changes.
• This will be followed by a 4-week period of protocol implementation. Trusts with their own protocol will be required to re-circulate their pre-existing protocol whilst trusts with no official protocol will be offered the King’s College Hospital Cardiac Surgical Pain Management Protocol (Table 1) for implementation. Following the implementation period, pain will be re-audited and data will be collected over a further 8-week period.

### 2.5 Stage 1: Preliminary survey

Students will also be asked to collect data on current pain management strategies, including whether their institution makes use of a pain management protocol. This will be done through identifying the senior cardiac anaesthetist and cardiac surgery matron, and asking them a series of questions directly (Table 3; Appendix 1). These questions have been approved by the cardiothoracic department at our institution.

**Table 3:** Details on current pain management guidelines.

1. Is there an official pain management protocol* for post cardiac surgery patients? * if there is a protocol this is to be obtained, please obtain it
2. How is pain routinely managed?
3. Have you identified from clinical experience patients with high levels of pain?
4a. Please describe the characteristics observed to be associated with higher levels of pain in further details below.
4b. Are patients with these characteristics managed differently?
5. How is pain measured? (Frequency of measurement, method/scale used, by whom)

### 2.6 Stage 2: Baseline audit

Students will be asked to record pain levels on 3 POD in 50 patients over a period of 8 weeks. Each student will be given step-by-step protocol to standardise data collection methods across all trusts and eliminate bias. Pain will be measured at any point on the first POD as it would be impractical otherwise. Data will be collected on pain scores at rest, moving and coughing on the first three postoperative days using a Likert numerical rating scale (0–10). Frequency of pain measurements by the nursing care team and administration of PRN analgesia will also be recorded. Details about the surgery including type of surgery, conduits used (if applicable) and cross-clamp time will be extracted from the operation notes. Data will also be collected on the patient’s age, gender, race, and risk factors for pain (opioid abuse, chronic pain and long-term analgesia use) from patient records as summarised in Table 4. This will enable a before and after comparison of protocol implementation. EUROScore will be collected from the lead consultant at each trust.

**Table 4:** Data collection.

**Part I only**
*Trust information*
- Current Pain Management Guidelines - Protocol Information • Analgesia: type, dose, route of information and duration • Stratification of patients (high- and low-risk)
**Part II & Part III**
*Demographic information*
- Patient age - Patient gender - Patient ethnicity
*Procedure information*
- Type of Surgery (CABG, AVR, combined) - EURO Score - Type of Conduits (including single or bilateral internal mammary harvesting) - Harvesting method (bridging, open, endoscopic) - Measure of risk of pain (high-risk or low-risk) - High-risk: previous chronic analgesic use, previous chronic pain, opioid abuse
*Medication*
- Pre-operative gabapentin (yes/no; if yes: dose, timing) - Intra-operative: type, dose, route of information and duration - Post-operative: type, dose, route of information and duration • Frequency and dose of PRN actual administration of analgesics
*Nursing care*
- Frequency of documented pain measurements per day
*Outcome variables*
- Pain scores on post-operative day 1–3 measured with Likert scale (0–10): • Rest • Moving • Coughing

### 2.7 Stage 3: Protocol implementation & re-audit following protocol implementation

Each student will then undertake a 4-week period of protocol implementation. Trusts with their own official protocol will be given the option to re-circulate their pre-existing protocol or to adapt the King’s College Hospital Cardiac Surgical Pain Management Protocol. Trust with no official protocol will be offered the KCH Protocol for implementation. Those who do not have a pre-existing protocol nor wish to adapt the KCH Protocol will not undertake any intervention or data collection in this 4-week period. Protocol implementation may include circulation of the protocol through departmental emails, flyers in relevant clinical areas (e.g. cardiac surgical wards) and presentations at department audit or clinical governance meetings.

Following the 4-week implementation period, all students will be asked to collect data (as per Table 4; Appendix 2) from a further 50 patients over a period of 8 weeks. Trusts without any official pain protocol at this stage will continue to be included in the re-audit data collection.

### 2.8 Data protection

All data will be anonymised locally and input into a preformed encrypted spreadsheet to be given to the study leads. No identifiable patient data are to leave the respective trust and all published data will be anonymised with respect to the patient and the trust. Individual trust data or consultant names will also not be published in an identifiable manner. All data will be analysed centrally by the study leads.

### 2.9 Variables

The independent variables include: risk of pain (high- and low-risk); age; time; and protocol implementation. The dependent variables are pain scores on a Likert Scale (0–10) measured at rest, movement and coughing, on postoperative days 1, 2 and 3. Controlled variables include EUROScore and gender.

### 2.10 Statistics

Power analysis will be done to calculate the minimum number of participants required for stage 3. A univariate and multivariate regression will be conducted on pain scores. Pain scores will be compared across trusts to elicit geographical variation and protocol-based variation, across individual factors (race, age and gender) to elicit demographic variation, across high- and low-risk groups to elicit the significance of risk of pain in developing pain, and comparison of pain across the 3 post-operative days. Continuous variables will be summarised using the mean and the standard deviation. Significance will be calculated, with p < 0.05 deemed to be statistically significant.

In order to assess the importance of use of a protocol pain levels will be compared between centres that do and do not use a protocol, using an independent samples t-test if data are normally distributed or Kruskal–Wallis if data are skewed. Protocols will also be analysed qualitatively for their content.

### 2.11 Dissemination

The work will be published in a peer-reviewed journal and presented at national and international meetings within the field of cardiothoracic surgery and anaesthesia. The work will be disseminated electronically and in print. Brief reports of the review and findings will be disseminated to interested parties through email and direct communication. The review aims to guide healthcare practice and policy.

## Ethical approval

Not applicable.

## Funding

None.

## Author contribution

Shruti Jayakumar – Study Design, Data Collection, Data Analysis, Writing

Mimi Borrelli – Study Design, Data Collection, Data Analysis, Writing

Zoka Milan – Study Design, Manuscript Revisions, Writing

Gudrun Kunst – Study Design, Manuscript Revisions, Writing

Donald Whitaker – Study Design, Manuscript Revisions, Writing

## Conflicts of interest

None.

## Guarantor

Shruti Jayakumar, Gudrun Kunst, Donald Whitaker.

## Research Registration Unique Identifying Number (UIN)

Not applicable.

## Figures and Tables

**Figure:**
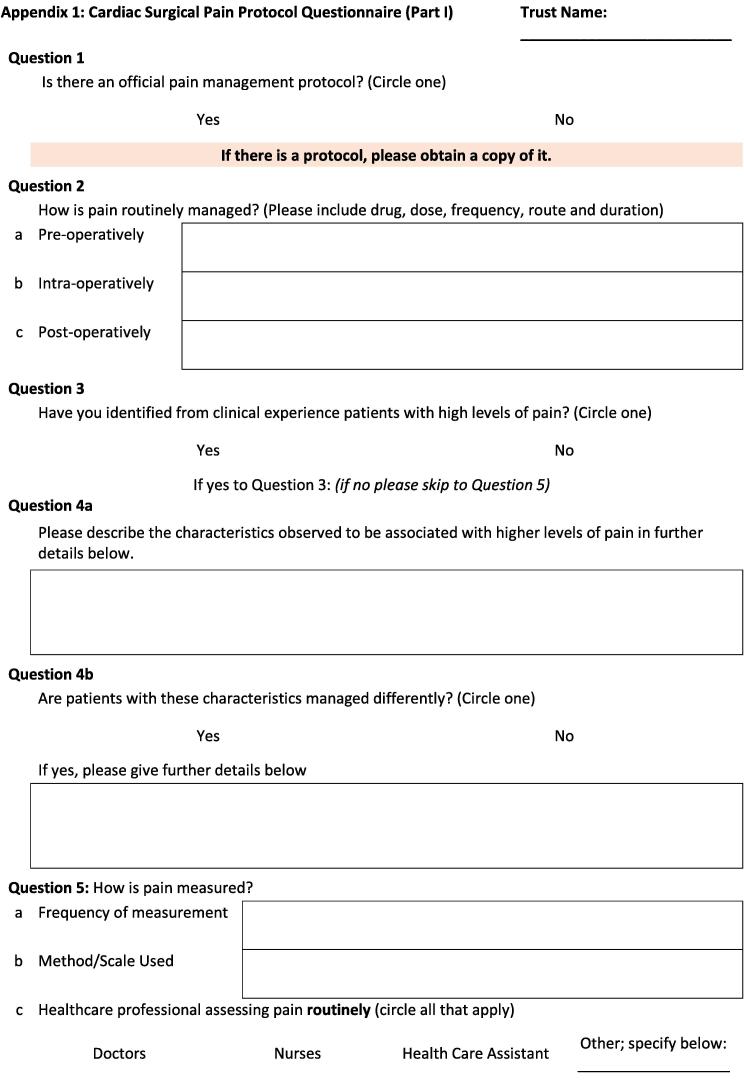


**Figure:**